# Attitudes and intentions toward prostate cancer screening among males in China: a qualitative study

**DOI:** 10.3389/fruro.2025.1698789

**Published:** 2025-12-09

**Authors:** Shutao Hao, Linlin Fang, Yanting Du, Jin Zheng, Yufeng Ou, Zhenghong Yu

**Affiliations:** 1 Department of Urology, Beijing Tongren Hospital, China Medical University (CMU), Beijing, China; 2 Department of Urology, Zhongshan Hospital, Fudan University, Shanghai, China; 3 Department of Urology, The First Hospital of China Medical University, Shenyang, China

**Keywords:** prostate cancer screening, theory of planned behavior, intention, behavior, qualitative study

## Abstract

**Purpose:**

To explore the factors influencing prostate cancer screening willingness among the Chinese male population.

**Methods:**

This is a qualitative study that adopted a phenomenological approach. Purposive sampling was used to recruit 21 males (aged 48–81 years, with a mean age of ~57 years) from the health examination center of a tertiary hospital in Shenyang, China. Semi-structured face-to-face interviews were verbatim transcribed and independently cross-checked by two Master’s-level researchers. Data analysis was performed using MaxQDA ver.10 software and followed Colaizzi’s phenomenological analysis steps.

**Results:**

Deductive analysis identified three core themes aligned with the Theory of Planned Behavior (TPB): Attitudes toward the Behavior (getting screened for the sake of family, primary prevention actions, meaningless, fear of the result, and don’t want family members to worry), subjective norm (family members, friends, and colleagues, and health professionals), and control beliefs (cost and insurance, limited understanding of the disease, no symptoms, unfamiliar with the PSA test, doubt screening, health examination package setting, and believe that cancer is incurable).

**Conclusions:**

The study highlights the complex and unique factors influencing willingness to undergo prostate cancer screening in China. The findings provide insights for developing targeted interventions to address the challenges of insufficient prostate cancer screening, particularly by enhancing health professional guidance and addressing financial barriers.

## 1 Introduction

Prostate cancer (PCa) is the second most commonly diagnosed cancer and the fifth leading cause of cancer-associated mortality in men globally (1). Its incidence is rising worldwide, with decreasing mortality rates in developed countries but increasing rates in developing countries (1). In China, prostate cancer’s incidence and mortality rates are 9.8 per 100,000 and 4.22 per 100,000, respectively (2, 3), highlighting prostate cancer as a significant health threat to Chinese males.

Early detection of prostate cancer is crucial for effective treatment and improved survival rates (4, 5). However, prostate cancer is frequently diagnosed at an advanced stage due to the absence of early clinical symptoms (6). Screening methods primarily include prostate-specific antigen (PSA) testing, digital rectal examination (DRE), and transrectal ultrasound-guided biopsy (TRUS), with PSA testing being the most prevalent due to its 92.3% sensitivity (7) and mortality reduction of 20%-27% (8, 9). Despite these benefits, screening rates vary, with developed countries like European countries and the United States having rates of 72.2% (10) and 54% (11), respectively, while only 37.9% of Chinese males are aware of PSA testing (12). This disparity is attributed to limited public knowledge, uneven healthcare resources, and cultural barriers (13–15), resulting in many Chinese males being diagnosed at advanced stages and missing the optimal treatment window.

**Figure 1:**
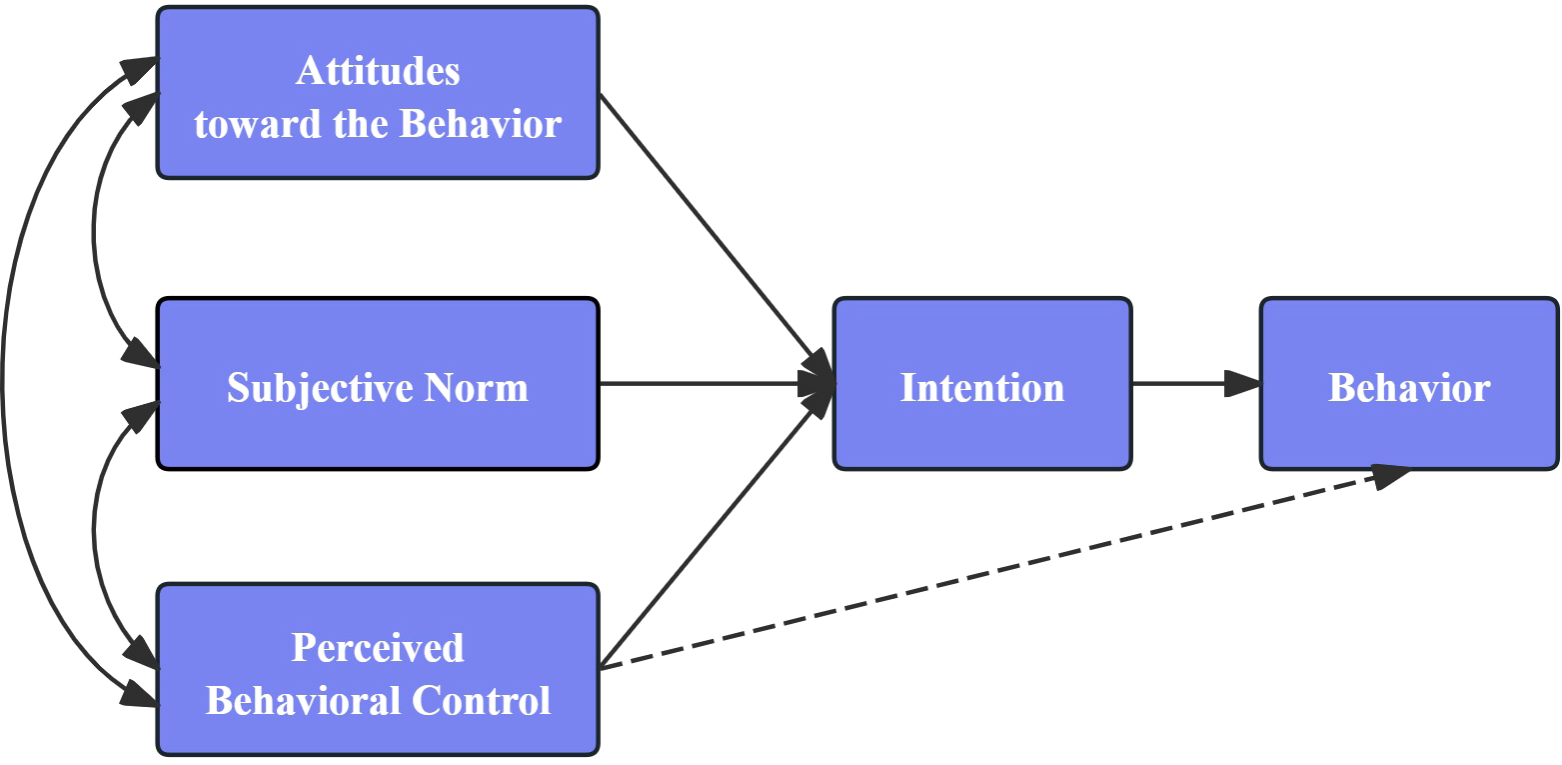
The theory of planned behavior (TPB) model.

The Theory of Planned Behavior(TPB) is a well-established model (16) that has been widely applied to the prediction of behaviors (17–19). According to the TPB model (see **Figure 1**), an individual’s behavioral intention is the most immediate predictor of their actions, and this intention is influenced by three key factors: attitude, subjective norms, and perceived behavioral control (16). It has been extensively applied in studies of various health behaviors, including reproductive decisions (20) and cancer surveillance (21). It has greater explanatory power than other models such as the health model, and social cognitive theory (22, 23). Consequently, this study employed in-depth interviews based on the TPB framework to investigate the factors affecting prostate cancer screening among Chinese males aged 40 and older.

While the TPB model is widely used in health behavior research, most studies have focused on Western populations, with limited qualitative research on Chinese males. Existing studies indicate that factors influencing prostate cancer screening are complex and varied, including individual health status, clinical symptoms, social relationships, demographic characteristics, and the knowledge of PSA screening (13–15). However, findings from these studies, which are predominantly based on Western populations, may not fully reflect the screening behaviors and influencing factors of Chinese males. Additionally, most research has employed quantitative methods, focusing on statistical analyses to explain screening behavior patterns, lacking in-depth exploration of the underlying social, cultural, and psychological mechanisms. This study aims to fill this gap by employing qualitative research methods based on the TPB model to examine attitudes, subjective norms, and perceived behavioral control regarding prostate cancer screening. By analyzing these factors, we seek to identify the main barriers and facilitators influencing prostate cancer screening behaviors among Chinese males.

## 2 Method

### 2.1 Study design and participants

This qualitative study adopted a phenomenological approach to explore the lived experiences and factors influencing prostate cancer screening willingness among Chinese males. The data collection was conducted from 3 March to 15 May 2021.

#### 2.1.1 Sampling and sample size

Purposive sampling was used to recruit participants from the health examination center of a Tertiary A hospital in Shenyang, China. As is standard practice in qualitative research, data saturation was used to determine the final sample size. The data collection was terminated when no new topics appeared (24). Finally, 21 males were included in the study, with a mean age of approximately 57 years (range: 48–81 years). Respondents’ characteristics are presented in **Table 1**. The interviewees were aged between 48 and 81 years, with a mean age of approximately 57 years old. Nearly half held a bachelor’s degree or higher.

**Table 1:** Characteristics of participants (*N* = 21).

Interviewee	Age (years)	Education status	Vocation
M1	54	High school	Self-employed
M2	55	Associate’s degree	Business management personnel
M3	57	Master’s degree	Retirement
M4	65	Bachelor’s degree	Retirement
M5	48	Bachelor’s degree	Business management personnel
M6	56	Bachelor’s degree	Business management personnel
M7	63	Bachelor’s degree	Retirement
M8	54	Bachelor’s degree	Civil servants
M9	63	Bachelor’s degree	Retirement
M10	58	Bachelor’s degree	Business management personnel
M11	55	Bachelor’s degree	Business management personnel
M12	50	Bachelor’s degree	Business management personnel
M13	76	Secondary vocational school	Carpenter
M14	57	Primary school	Farmer
M15	81	Associate’s degree	Retirement
M16	63	Junior high school	Self-employed
M17	44	Associate’s degree	Self-employed
M18	52	Master’s degree	Engineer
M19	46	Bachelor’s degree	Accountant
M20	50	Bachelor’s degree	Self-employed
M21	58	Doctor’s degree	Professor

Interviewee number: M1˜M21.

#### 2.1.2 Inclusion and exclusion criteria

Inclusion criteria: 1) Aged 40 years or older (as prostate cancer screening guidelines indicate that routine PSA screening is not recommended for males younger than 40 years or those with a life expectancy of less than 10–15 years (25)); 2) Undergoing routine or pre-employment health screenings at the hospital.

Exclusion criteria: 1) History of prostate cancer; 2) Current diagnosis of prostate cancer; 3) Individuals seeking screening services independently.

### 2.2 Data collection

#### 2.2.1 Instrument structure

The primary data collection instrument was a semi-structured interview outline (**Table 1**). The outline was developed based on the four core dimensions of the Theory of Planned Behavior (TPB): behavioral intention, subjective norms, perceived behavioral control, and attitude. The initial draft included 12 questions designed to probe beliefs and attitudes related to screening.

#### 2.2.2 Instrument validity and credibility

To ensure the content validity of the interview guide, the initial outline was revised after consulting two nursing researchers and one urology clinician. This process ensured the questions were relevant to the clinical and theoretical focus. The final interview questions are presented in **Table 2**.

**Table 2:** The semi-structured interview form.

Concepts in the conceptual framework	Example interview questions
Behavior intention	Are you involved in prostate cancer screening?
Subjective norm	Who will influence your prostate cancer screening? Who are the most likely factors to affect prostate cancer screening decisions? Where do you find information on prostate cancer screening?
Perceived behavioral control	What motivates you to get screened for prostate cancer? What prevents you from participating in prostate cancer screening?
Behavior attitude	What do you see as the benefits of prostate cancer screening? Do you think prostate cancer screening is necessary? why or why not? What are the risks or disadvantages of prostate cancer screening?

### 2.3 Procedures

Interview Setting and Data Gathering. Data were collected through semi-structured, face-to-face, in-depth interviews. Interviews were conducted by two trained authors. Chinese was the language of communication. Each interview was voice-recorded and lasted between 30 and 60 minutes (37 minutes minimum, 54 minutes maximum).

The interviews were conducted by two female authors, both Master’ s-level nursing researchers specializing in Urology. The gender difference between the interviewers and male participants warranted careful consideration, as some participants might have been reluctant to discuss sensitive, gender-specific health topics such as prostate cancer screening. However, the researchers’ positionality as female nursing specialists might have created a perceived neutral environment, which potentially encouraged male participants to express emotional or relational concerns (e.g., family worries) more openly. Their specialized urology research background provided expertise to probe deeply into related themes but was managed through strict adherence to the semi-structured interview guide to minimize the potential imposition of clinical judgment. All potential influences, including the interviewer–participant gender dynamic, were continuously examined during peer debriefing sessions to uphold methodological rigor.

### 2.4 Data analysis

To ensure data integrity and validity, all audio recordings were verbatim transcribed within 24 hours by a professional transcriber. Two researchers (with 2 years of qualitative data analysis experience) independently cross-checked the transcripts against the recordings, marking discrepancies and resolving them through joint review of the original audio - this process ensured a final error rate of <1%. Data were entered into MaxQDA ver.10 software, with a daily backup stored on a password-protected hospital research server and access restricted to 3 core team members.

Data analysis followed Colaizzi’s phenomenological analysis steps (26). This analysis included six steps: (1) Repeatedly listening to each recording and re-reading transcribed interview to gain a comprehensive understanding of each interviewee’s perspectives; (2) Extracting significant statements from the transcripts; (3) Assigning meaning to the extracted units; (4) Grouping similar units into categories, themes, and subthemes; (5) Providing comprehensive descriptions of the extracted categories; (6) Creating a basic paradigm of the subject under study based on the extracted categories.

### 2.5 Rigor and trustworthiness

Lincoln and Guba’s criteria (27) were used to evaluate the rigor and trustworthiness of this study. The rigor was established across the dimensions of credibility, dependability, and transferability.

#### 2.5.1 Credibility

Credibility, or the confidence in the truth of the findings, was established through several techniques.

#### 2.5.2 Investigator triangulation

The two interviewers, both master’s-level researchers specializing in Urology, independently verified the transcripts against the recordings and ensured consistency throughout the coding process. Consensus on coding and thematic classification was achieved within the core research team, thereby ensuring that the findings were not reliant on any single researcher’s interpretation.

#### 2.5.3 Peer debriefing

The research team held regular debriefing sessions with an experienced qualitative researcher who was not directly involved in data collection. These sessions involved reviewing the coding frameworks, emerging themes, and potential researcher biases to critically examine underlying assumptions and interpretations, thereby providing external validation of the analytic process.

#### 2.5.4 Clarification on member checking

The theoretical procedures of Colaizzi’s method include participant verification, commonly referred to as member checking. However, due to logistical constraints related to participant traceability within the health examination center context, formal member checking could not be performed. Consequently, neither complete transcripts nor summarized thematic results were returned to participants for validation. Instead, credibility was rigorously established through the combined strengths of investigator triangulation and peer debriefing.

#### 2.5.5 Dependability, audit trail, and transferability

Dependability refers to the stability and consistency of findings over time and across researchers.

#### 2.5.6 Audit trail

A comprehensive record of the research process was maintained throughout the study, including all voice recordings, transcripts, and field notes, thereby demonstrating the logical progression of the research.

#### 2.5.7 Detailed methodology

A clear and comprehensive description of the data collection and analysis procedures, based on Colaizzi’s phenomenological steps, was documented to ensure methodological transparency.

#### 2.5.8 Transferability

To enhance transferability, thick descriptions were provided of the methodology, study context (a tertiary A hospital in Shenyang, China, serving a diverse population), and participants’ characteristics (e.g., age, education, occupation), as detailed in **Table 2**. The findings section also incorporates numerous direct quotations to authentically represent participants’ voices.

### 2.6 Ethical considerations

This research was approved by the Medical Ethical Committee of The First Affiliated Hospital of China Medical University (Ref. 360/2020 on 4 January 2021). Prior to the interview, participants received information about the study’s aim and content, and informed consent was obtained from all participants. Participants were explicitly informed of their right to withdraw at any time without consequences, thus ensuring the voluntariness of their participation. To safeguard participant privacy and ensure anonymity, all participants were assigned identification codes (M1–M21). The corresponding audio recordings and transcripts were stored on a password-protected device with access limited to the core research team, thereby ensuring confidentiality. All study procedures involving human participants complied with the ethical standards of the institutional and national research committees and adhered to the 1964 Helsinki Declaration and its subsequent revisions (most recently, Brazil 2013).

## 3 Results

**Figure 2:**
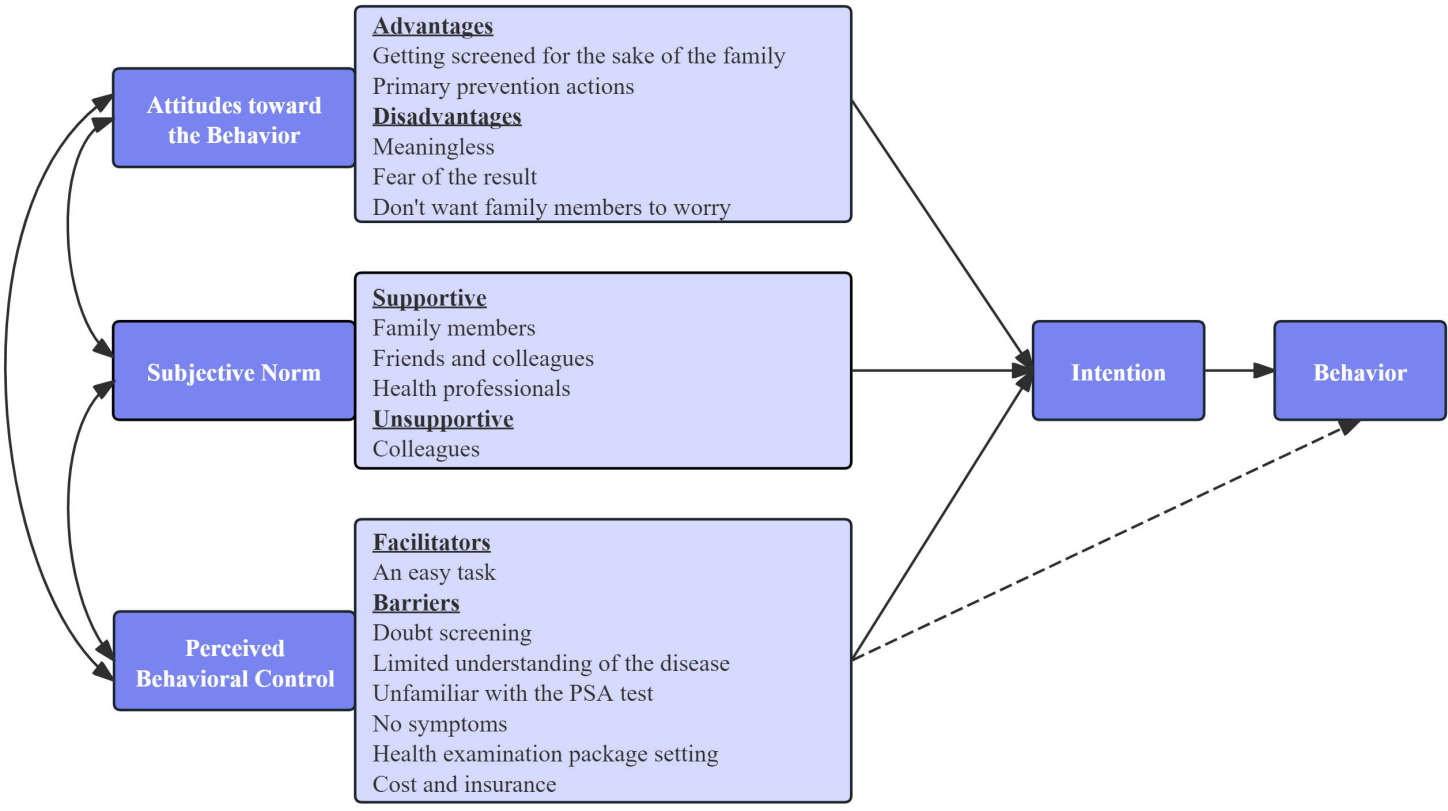
Thematic Framework of factors influencing prostate cancer screening behavior among Chinese males (based on the Theory of Planned Behavior).

The thematic analysis revealed three core categories aligning with the Theory of Planned Behavior (TPB): Attitudes toward the Behavior, Subjective Norm, and Perceived Behavioral Control. These themes and their associated subthemes are summarized in **Table 3** and illustrated in **Figure 2**.

**Table 3:** Thematic analysis: TPB category, themes, and exemplar supporting quotes on prostate cancer screening willingness among Chinese males.

TPB Categor	Theme	Subtheme	Exemplar supporting quote
Attitudes toward the Behavior	Beliefs in Advantages	Getting screened for the sake of the family	“At my age, the pressure is sort of maximized as I have a big family to support, hence I pay great attention to my health. I hope to diagnose any disease as early as possible.”[M19, 46, Bachelor’s degree, Accountant]
		Primary prevention actions	“Nowadays, medicine is advanced and some cancers can be cured with the help of early diagnosis, so I think prostate cancer could be cured.”[M4, 65, Bachelor’s degree, Retirement]
	Beliefs in Disadvantages	Meaningless	“I’m already 80 years old, and there’s no need for treatment even if cancer is detected … It would only make me anxious.” [M15, 81, Associate’s degree, Retirement]
		Fear of the result	“Many people, especially with those low payment, are scared by health examination.” [M18, 52, Master’s degree, Engineer]
		Don’t want family members to worry	“I don’t want my children and relatives to receive information about my illness cause them worry about me.” [M15, 81, Associate’s degree, Retirement]
Subjective Norm	Normative beliefs - supportive	Family members	“My brother was died of prostate cancer, so my mother ask me to do it.” [M17, 44, Associate’s degree, Self-employed]
		Friends and colleagues	“If your friends and colleagues are suffering from prostate cancer, you will follow their advice and take a screening.” [M8, 54, Bachelor’s degree, Civil servants]
		Health professionals	“If a doctor advises me to take a prostate cancer screening, I would not hesitate to take one.” [M2, 55, Associate’s degree, Business management personnel]
	Normative beliefs - unsupportive	Colleagues	“My coworkers said that health check-ups are a waste of money since they only catch serious illnesses, so what’s the point?” [M9, 63, Bachelor’s degree, Retirement]
Perceived Behavioral Control	Control beliefs - facilitators	An easy task	“Serum test of PSA? Just take a tube of blood. It’s acceptable for me.”[M4, 65, Bachelor’s degree, Retirement]
	Control beliefs - barriers	Cost and insurance	“Ordinary people can’t afford cancer screening at all (excitedly)!” [M14, 57, Primary school, Farmer]
		Limited understanding of the disease	“I often hear about breast cancer and cervical cancer on television, but prostate cancer is rarely mentioned.”[M5, 48, Bachelor’s degree, Business management personnel]
		No symptoms	“I won’t do it actively because I don’t have any symptoms.” [M3, 57, Master’s degree, Retirement]
		Unfamiliar with the PSA test	“The health examination package provided by the company usually includes abdominal color Doppler ultrasound examination, which should be able to detect prostate problems.” [M1, 54, High school, Self-employed]
		Doubt screening	“Many health examinations are merely a formality and the likelihood of diagnosing a disease is quite low.” [M5, 48, Bachelor’s degree, Business management personnel]
		Health examination package setting	“Therefore, the male package should also include a prostate cancer screening.” [M2, 55, Associate’s degree, Business management personnel]
		Believe that cancer is incurable	“Getting cancer means waiting for death. What’s the point of getting any tests?” [M10, 58, Bachelor’s degree, Business management personnel]

### 3.1 Attitudes toward the behavior

#### 3.1.1 Beliefs in advantages

##### 3.1.1.1 Getting screened for the sake of the family

Nine participants (9/21) take cancer screening as a part of family responsibility, as they believe that screening can help clarify their health conditions.

“At my age, the pressure is sort of maximized as I have a big family to support, hence I pay great attention to my health. I hope to diagnose any disease as early as possible.” [M19, 46, Bachelor’s degree, Accountant].

##### 3.1.1.2 Primary prevention actions

Four participants (4/21) believed that prostate cancer screening is helpful, as early diagnosis of cancer leads to improved prognosis and reduced complications.

“Nowadays, medicine is advanced, and some cancers can be cured with the help of early diagnosis, so I think prostate cancer could be cured.” [M4, 65, Bachelor’s degree, Retirement].

“I haven’t had any health examination in the past two years, and this time I chose a package with a wide range of items, including cardiovascular and cerebrovascular ones, and tumor screening. After all, I am growing old.” [M17, 44, Associate’s degree, Self-employed].

“I choose different items every year according to my conditions. If there is any abnormality, I’ll go for further examination.” [M12, 50, Bachelor’s degree, Business management personnel].

#### 3.1.2 Beliefs in disadvantages

##### 3.1.2.1 Meaningless

Fifteen participants (15/21) believe cancer screening is meaningless because there is nothing to be done except waiting for death.

“All cancers were identified in the late stage … You can do nothing but wait for death.” [M10, 58, Bachelor’s degree, Business management personnel].

“I’m already 80 years old, and there’s no need for treatment even if cancer is detected … It would only make me anxious.” [M15, 81, Associate’s degree, Retirement].

##### 3.1.2.2 Fear of the result

Twelve participants (12/21) fear being diagnosed with cancer, believing that avoiding screenings means they won’t have to face the reality of having cancer. This avoidance is often fueled by the observed psychological and financial distress that a cancer diagnosis causes others, which they use to rationalize their own preventative inaction.

“Many people, especially with those low payments, are scared of health examinations. Such a disease can cause serious psychological effects!” [M18, 52, Master’s degree, Engineer].

##### 3.1.2.3 Don’t want family members to worry

Seventeen participants (17/21) avoided screening to prevent their family members from becoming concerned.

“I don’t want my children and relatives to receive information about my illness cause it will worry them about me.” [M15, 81, Associate’s degree, Retirement].

### 3.2 Subjective norm

Their subjective norms about screening Influences by significant others in the lives of the participants. The “significant others” were principally family members, friends, colleagues, and health professionals.

#### 3.2.1 Normative beliefs -supportive

##### 3.2.1.1 Family members

Among the participants, three males (3/21) who reported a family history of cancer were strongly aware of the impacts of cancer on their health and quality of life.

“My family has a history of cancer, so I take annual cancer screening.” [M20, 50, Bachelor’s degree, Self-employed].

“My brother died of prostate cancer, so my mother asked me to do it (prostate cancer screening).” [M17, 44, Associate’s degree, Self-employed].

##### 3.2.1.2 Friends and colleagues

The attitudes and behavior of the males’ friends and colleagues also had an influence on their behavior. Three participants (3/21) indicated that the advice of peers would prompt them to consider screening.

“If your friends and colleagues are suffering from prostate cancer, you will follow their advice and take a screening.” [M8, 54, Bachelor’s degree, Civil servants].

##### 3.2.1.3 Health professionals

Notably, five participants (5/21) emphasized that the health professionals at the hospital were the most authoritative and important influences on their intention for prostate cancer screening.

“If a doctor advises me to take a prostate cancer screening, I would not hesitate to take one.” [M2, 55, Associate’s degree, Business management personnel].

“Suggestions from friends or colleagues would have some influence. However, the doctor’s advice and my conditions would be determining.” [M6].

Despite these responses, most of the men interviewed in the present study reported that no-one had reminded them about the PSA testing.

“How are we supposed to know about this test? It is the doctor’s responsibility to inform us, but no one told me (about the likelihood of prostate issues).” [M6, 56, Bachelor’s degree, Business management personnel].

#### 3.2.2 Normative beliefs -unsupportive

##### 3.2.2.1 Colleagues

Three participants (3/21) reported that their colleagues held an unsupportive stance toward health check-ups, influencing their own skepticism.

“My coworkers said that health check-ups are a waste of money since they only catch serious illnesses, so what’s the point?” [M9, 63, Bachelor’s degree, Retirement].

### 3.3 Perceived behavioral control

#### 3.3.1 Control beliefs -facilitators

##### 3.3.1.1 An easy task

Four individuals (4/21) are willing to undergo screening because they find it acceptable and straightforward.

“Serum test of PSA? Just take a tube of blood. It’s acceptable for me.” [M4, 65, Bachelor’s degree, Retirement].

#### 3.3.2 Control beliefs – barriers

##### 3.3.2.1 Cost and insurance

In this study, ten participants (10/21), including both insured and uninsured individuals, expressed that cost and insurance were major obstacles.

“If the cost is reasonable and covered by medical insurance, I would like to undergo one.” [M3, 57, Master’s degree, Retirement].

“For prostate cancer screening, I believe that the acceptance of screening is related to personal awareness and cost.” [M6, 56, Bachelor’s degree, Business management personnel].

“Ordinary people can’t afford cancer screening at all (excitedly)!” [M14, 57, Primary school, Farmer].

“You don’t understand the mentality of the elderly. We would never go for a health examination unless it were free.” [M15, 81, Associate’s degree, Retirement].

##### 3.3.2.2 Limited understanding of the disease

Seven participants (7/21) stated that they had never heard of prostate cancer. Limited information about prostate cancer in the media restricts their knowledge about prostate cancer and its screening.

“I often hear about breast cancer and cervical cancer on television, but prostate cancer is rarely mentioned. I don’t think most citizens are aware of or even notice this disease.” [M5, 48, Bachelor’s degree, Business management personnel].

“I am a regular listener to health radio, but there is little information about it (prostate cancer). I have heard about prostatitis and prostatic hyperplasia, though.” [M9, 63, Bachelor’s degree, Retirement].

“Prostate cancer is not as common as breast cancer or stomach cancer.” [M21, 58, Doctor’s degree, Professor].

##### 3.3.2.3 No symptoms

For nine participants (9/21), the absence of symptoms leads them to avoid screening.

“I have some problems with the prostate. Although I am supposed to have a prostate cancer examination. I won’t do it actively because I don’t have any symptoms.” [M3, 57, Master’s degree, Retirement].

##### 3.3.2.4 Unfamiliar with the PSA test

Fourteen participants (14/21) expressed a lack of understanding regarding the PSA test. Although most health examination centers have listed the serum PSA test as a diagnostic item for prostate cancer screening, few males notice this item, let alone fully understand it.

“The health examination package provided by the company usually includes abdominal color Doppler ultrasound examination, which should be able to detect prostate problems.” [M1, 54, High school, Self-employed].

“The risks of the PSA test are a bit confusing for me, because I have undergone blood tests for other items without any reported problems.” [M3, 57, Master’s degree, Retirement].

“I have done a full PET-CT, and the reports are normal, so I don’t know why I have to do prostate cancer screening.” [M16, 63, Junior high school, Self-employed].

##### 3.3.2.5 Doubt screening

Eleven participants (11/21) are skeptical about the effectiveness of screening. They argue that many health examinations, including prostate cancer screenings, may be mere formalities with low diagnostic value.

“Many health examinations are merely a formality, and the likelihood of diagnosing a disease is quite low.” [M5, 48, Bachelor’s degree, Business management personnel].

“Some health examination results are highly predictable, such as atherosclerosis, but when it comes to prostate cancer … I’m afraid not.” [M13, 76, Secondary vocational school, Carpenter].

##### 3.3.2.6 Health examination package setting

Indeed, nine participants (9/21) claimed that cancer screenings should be included in the health examination package.

“Health examinations provided by our companies are standardized. Fortunately, the female package includes a gynecological examination. Therefore, the male package should also include a prostate cancer screening.” [M2, 55, Associate’s degree, Business management personnel].

##### 3.3.2.7 Believe that cancer is incurable

Twelve participants (12/21) perceive cancer as formidable, perceiving its treatment process as arduous and with limited prognostic benefits in terms of life extension.

“Getting cancer means waiting for death. What’s the point of getting any tests?” [M10, 58, Bachelor’s degree, Business management personnel].

## 4 Discussion

Prostate cancer represents a major health challenge for males worldwide, with early screening playing a critical role in reducing mortality and improving diagnostic and treatment outcomes (28). The findings of this study indicate a general lack of awareness about prostate cancer screening among Chinese males. This lack of awareness is reflected in skepticism about the utility of screening, concerns about potential results, and a negative perception of cancer. Over half of the sample (12/21) perceived cancer as incurable, directly contributing to the skepticism. This is consistent with findings from several studies. This is consistent with findings from several studies (29, 30). Furthermore, there is a significant gap in knowledge regarding Prostate-Specific Antigen (PSA) testing among Chinese males, which adversely affects their willingness to participate in screening programs. This knowledge gap is evidenced by the fact that 14 participants (14/21) expressed unfamiliarity with the PSA test. Contributing factors include educational and economic barriers that limit understanding of the benefits of cancer screening (31, 32). In contrast, Western countries generally emphasize the importance of early detection and treatment, supported by comprehensive insurance systems that mitigate financial barriers and enhance public education about screening practices (33). These approaches contribute to higher participation rates and improved health outcomes (34).

This study highlights several key factors influencing Chinese males’ willingness to undergo prostate cancer screening, including social support, economic status, awareness, health literacy, and insurance coverage. These elements create a complex decision-making landscape that impacts screening participation. Social support plays a crucial role, as attitudes and recommendations from family, friends, colleagues, and healthcare professionals significantly affect males’ screening decisions. Men with a family history of cancer often view screening more positively, though concerns about causing emotional stress to their families if diagnosed with cancer also arise. This dual role of social support, especially within the Chinese cultural context, underscores the importance of family in decision-making. Notably, five participants (5/21) specifically cited healthcare professionals as the most authoritative influence on their screening intention. As Rezaei et al. (35)suggested, enhancing health education for both patients and their families can improve screening attitudes and knowledge. Thus, leveraging social support from families, peers, and healthcare professionals is vital for encouraging early screening among high-risk populations.

Economic factors, including screening costs and insurance coverage, significantly impact the willingness to undergo prostate cancer screening. Ten participants (10/21), representing nearly half of the sample, viewed prostate cancer screening as costly and indicated a higher willingness to participate if the expenses were covered by health insurance. Currently, most health insurance plans in China do not cover prostate cancer screening, adding a financial burden and limiting access to screening (36). This economic pressure is particularly pronounced among elderly males, who often feel they will not participate unless the screening is free. This lack of coverage, combined with the finding that nine participants (9/21) noted the PSA test’s omission from standard health packages, suggests major systemic barriers to access. Smith et al. (15)found similar results, highlighting the need to target elderly populations for increased screening awareness. In contrast, in economically developed countries, screening costs are frequently covered by government or insurance programs, improving accessibility. To enhance participation among low-income and elderly groups, policymakers should consider incorporating prostate cancer screening into routine health check-ups and ensuring insurance coverage through targeted policy interventions.

Lack of awareness and low health literacy are significant factors contributing to the low participation rate in prostate cancer screening among Chinese males. Seven participants (7/21) reported limited knowledge about prostate cancer, and 11 participants (11/21) expressed active doubt about the effectiveness of screening, equating a diagnosis with a death sentence. This fear and misunderstanding highlight the need for better public education on prostate cancer in China (35). Additionally, a high proportion of respondents (14/21) indicated that they had never heard of PSA testing. This lack of awareness not only impacts understanding of prostate cancer screening but also causes many males to overlook this crucial test during their regular health check-ups. Enhancing health literacy can improve participation in cancer screening (37), as studies show that individuals with higher health literacy are more likely to engage in screening programs, leading to reduced cancer mortality rates (37, 38). Therefore, future efforts in screening and early diagnosis should prioritize enhancing public awareness of the “three early” concepts (early detection, early diagnosis, and early treatment) (39). This can be achieved by developing cancer screening education platforms and organizing awareness campaigns to improve self-awareness of early detection among high-risk groups, ultimately promoting changes in preventive behaviors (40).

## 5 Strengths and limitations

This study provides several notable strengths. Methodologically, the use of Colaizzi’s phenomenological approach allowed for an in-depth, rich exploration of the complex factors influencing prostate cancer screening willingness among Chinese males. Furthermore, the study is theoretically grounded, utilizing the Theory of Planned Behavior (TPB) framework to systematically identify the underlying psychological, social, and control factors. Critically, it addresses a significant research gap by focusing specifically on the unique context of Chinese males, where existing literature is scarce. However, several limitations should be acknowledged. Primarily, the small sample size (n=21), despite achieving data saturation, restricts the generalizability of the findings, preventing them from being fully representative of a broader Chinese male population. Furthermore, the recruitment site, a single tertiary hospital’s health examination center, introduces a potential selection bias, as participants’ views may not fully reflect those of males who have never undergone a health check-up. Beyond these sample limitations, the qualitative nature of the study meant that there was no standardized protocol or patient outcome data collected, limiting the ability to draw causal conclusions. The design also did not include an exploration of the specific reasons behind certain deficiencies (e.g., why health professionals do not routinely prompt screening), which warrants further investigation. Future research should involve a larger and more diverse sample from different socioeconomic backgrounds to improve generalizability and external validity.

## 6 Conclusions

In summary, this study revealed several factors that influence Chinese males’ willingness to undergo prostate cancer screening, including social support, economic level, lack of awareness, screening costs, and insurance coverage. While some factors are common across countries, China’s unique social, cultural, and healthcare contexts pose specific challenges. These insights suggest that tailored interventions, considering the distinct social, economic, and cultural conditions of various regions, are essential for improving global participation in prostate cancer screening and promoting early detection and treatment.

### 6.1 Clinical implications

The study suggests that medical institutions should regularly promote prostate cancer prevention and that health examiners should actively serve as educators before and after screening. Health examination centers should enhance risk assessment and support for key screening targets. Additionally, insurance reimbursement for cancer screening can increase participation in China. To standardize screening, community-based early detection through a national multi-center study and a comprehensive management plan for high-risk groups are recommended, along with establishing a nationwide prostate cancer database.

## Data Availability

The raw data supporting the conclusions of this article will be made available by the authors, without undue reservation.
