# Correction: Attitudes and intentions toward prostate cancer screening among males in China: a qualitative study

**DOI:** 10.3389/fruro.2025.1764430

**Published:** 2026-01-02

**Authors:** Shutao Hao, Linlin Fang, Yanting Du, Jin Zheng, Yufeng Ou, Zhenghong Yu

**Affiliations:** 1 Department of Urology, Beijing Tongren Hospital, China Medical University (CMU), Beijing, China; 2 Department of Urology, Zhongshan Hospital, Fudan University, Shanghai, China; 3 Department of Urology, The First Hospital of China Medical University, Shenyang, China

**Keywords:** prostate cancer screening, theory of planned behavior, intention, behavior, qualitative study

Author “Linlin Fang” was erroneously omitted as “equal contributing author”.

The original version of this article has been updated.

